# Preventive evidence into practice (PEP) study: implementation of guidelines to prevent primary vascular disease in general practice protocol for a cluster randomised controlled trial

**DOI:** 10.1186/1748-5908-8-8

**Published:** 2013-01-18

**Authors:** Mark F Harris, Jane Lloyd, John Litt, Mieke van Driel, Danielle Mazza, Grant Russell, Jane Smith, Chris Del Mar, Elizabeth Denney-Wilson, Sharon Parker, Yordanka Krastev, Upali W Jayasinghe, Richard Taylor, Nick Zwar, Jinty Wilson, Helen Bolger-Harris, Justine Waters

**Affiliations:** 1 Centre for Primary Health Care and Equity, University of New South Wales, Kensington, NSW, 2052, Australia; 2 Discipline of General Practice, Flinders University, Adelaide, Australia; 3 Discipline of General Practice, University of Queensland, St Lucia, QLD, Australia; 4 School of Primary Health Care, Monash University, Melbourne, Australia; 5 Faculty of Health Sciences and Medicine, Bond University, Robina, QLD, Australia; 6 Faculty of Health, University of Technology Sydney, Ultimo, NSW, 2007, Australia; 7 Ethics Secretariate, University of Technology Sydney, Ultimo, NSW, 2007, Australia; 8 School of Public Health and Community Medicine, University of New South Wales, Kensington, NSW, 2052, Australia; 9 National Heart Foundation of Australia, Melbourne, Australia; 10 Royal Australian College of General Practitioners, Melbourne, Australia; 11 BUPA Foundation, Sydney, Australia

**Keywords:** Primary care, Family medicine, Guidelines, Preventive care, Cardiovascular disease

## Abstract

**Background:**

There are significant gaps in the implementation and uptake of evidence-based guideline recommendations for cardiovascular disease (CVD) and diabetes in Australian general practice. This study protocol describes the methodology for a cluster randomised trial to evaluate the effectiveness of a model that aims to improve the implementation of these guidelines in Australian general practice developed by a collaboration between researchers, non-government organisations, and the profession.

**Methods:**

We hypothesise that the intervention will alter the behaviour of clinicians and patients resulting in improvements of recording of lifestyle and physiological risk factors (by 20%) and increased adherence to guideline recommendations for: the management of CVD and diabetes risk factors (by 20%); and lifestyle and physiological risk factors of patients at risk (by 5%). Thirty-two general practices will be randomised in a 1:1 allocation to receive either the intervention or continue with usual care, after stratification by state. The intervention will be delivered through: small group education; audit of patient records to determine preventive care; and practice facilitation visits adapted to the needs of the practices. Outcome data will be extracted from electronic medical records and patient questionnaires, and qualitative evaluation from provider and patient interviews.

**Discussion:**

We plan to disseminate study findings widely and directly inform implementation strategies by governments, professional bodies, and non-government organisations including the partner organisations.

## Background

Cardiovascular disease (CVD) represents a substantial and increasing portion of healthcare expenditure and practitioner workload [1]. It is estimated that 9 in 10 adult Australians have at least one risk factor for CVD [2]. Behavioural risk factors include smoking, nutrition, alcohol, physical activity, and being overweight or obese. The physiological risk factors include high blood pressure and dyslipidaemia [3].

Primary care providers are well placed to help reduce the incidence of CVD. General practitioners provide clinical services to approximately 88% of Australians each year and are in an ideal position to screen for risk factors and provide brief interventions including advice about behavioural risk factors as well as medications [1]. However, there are numerous barriers to implementation at the patient, practitioner, practice, and system levels [4-7].

The Australian Government Department of Health and Ageing, the National Health and Medical Research Council, the National Heart Foundation of Australia, The Royal Australian College of General Practitioners, The Cardiac Society of Australia and New Zealand and the National Vascular Disease Prevention Alliance have published guidelines that address the major behavioural and physiological risk factors for vascular disease. These guidelines synthesise the evidence for preventing CVD and provide clear messages for primary care providers on what targets to aim for and what strategies might be best employed at the patient provider interaction.

**Figure 1:**
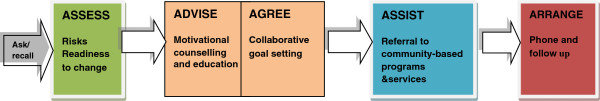
The 5As conceptual model.

A number of these guidelines refer to the 5As framework, which describes how general practice staff can intervene to prevent CVD [8]. This framework describes the pathway and informs the role of different providers and services in preventive care (Figure 1).

The guidelines have been widely disseminated and well received. However implementation requires more than dissemination [9], it requires a tailored approach to generate change in clinicians behaviour [10], and there exists evidence of gaps between the guideline recommendations and their implementation in Australian general practice [7,11,12]. Some of the reasons for the failure to implement prevention guidelines are related to the complexity of guideline recommendations and patient, practitioner, and practice barriers [7,13], including lack of capacity to provide brief interventions or refer for more intensive education and support [14,15]. Patients’ understanding of prevention is variable: they lack knowledge about what preventive care is what is relevant to them, and there is a tendency for patients with low health literacy and education attainment to be less likely to ask for, and therefore to receive, preventive care [16,17]. The culture of individual practices, their openness to change, and the number and experience of providers, all influence the ability to implement preventive guideline recommendations [18].

The Preventive Evidence into Practice (PEP) study is a partnership between New South Wales, Flinders, Monash, Bond, and Queensland Universities, the Royal Australian College of General Practitioners (RACGP), the National Heart Foundation of Australia (NHFA), and the BUPA Foundation. The PEP study is a national, cluster randomised control trial of an intervention designed to support general practices to implement the recommendations of evidence-based clinical management guidelines for the prevention of CVD in general practice among patients aged 40 to 69 years.

This study aims to evaluate the impact of the PEP intervention on: The behaviour of doctors and nurses in general practice in assessing and recording risk factors and providing interventions to address these; and patient behavioural and physiological risk factors.

We hypothesise that, for patients aged 40 to 69 years, the PEP intervention measured at the practice level over 12 months will improve: by 20% recording of behavioural and physiological risk factors; by 20% the adherence to the recommendations of guidelines for the management of these risk factors; and by 5% the lifestyle and physiological risk factors of patients with the risk factors.

## Methods

### Study design

The study is a cluster randomised controlled trial conducted in general practices in four states. This design was chosen because the primary intervention will be at the practice level and outcomes will be measured at the patient level.

### Randomisation

Practices will be randomly assigned to intervention and late intervention (control) groups after stratification into blocks by state and practice size—i.e., the number of general practictioners (GPs) in a practice—using a computer-generated randomization list. Randomisation will be conducted centrally, after completion of the baseline general practice and practice nurse (PN) surveys, by one of the investigators, a statistician not involved in the data collection or intervention (UJ). Results of the randomisation will communicated to the project management committee prior to the commencement of the intervention.

### Setting

The study is being conducted in general practices in four primary care organisations (Medicare Locals) in urban areas during 2012 and 2013.

### The intervention

#### Intervention development

The process for intervention development broadly followed the framework for design of complex interventions [19]. In order to inform the development of the PEP intervention we initially conducted a review of the literature [20]. This identified effective strategies for implementation of guidelines, including establishing small group education sessions with patients, clinician prompts and decision aids, audit and feedback, and external facilitation [21]. Educational interventions that are interactive, provided feedback to participants, include an objective assessment of education needs and involve small groups are more likely to be effective [22,23]. Small group interventions are most effective because they combine evidence-based material with peer influence [24]. Audit and feedback can be effective in providing more preventive care [25]. The variation of effect can be explained by the different ways in which audits are conducted and how feedback is provided. External facilitation has been shown to be effective in improving preventive care [26]. Facilitation is usually included as part of a multifaceted intervention that includes auditing medical records.

We then conducted a mixed method study involving eight Sydney based general practices which included qualitative interviews with eight staff from two divisions of general practice, one allied health provider, eight GPs, four PNs, three practice managers and 24 patients. We also conducted a clinical audit of medical records in the eight practices for CVD preventive activities (for 2,409 patients aged 40 to 69 years). The findings from this, and the literature review, were discussed at a workshop involving the investigators and partners and external stakeholders including consumers, professional organisational representatives, and policy makers. The intervention was then piloted in three Sydney general practices and evaluated through audio recording of facilitation visits and interviews with practice staff before and after the intervention. The analysis resulted in changes to the audit feedback to practices, the program of practice visits, and the resources provided to practices. Following the pilot a facilitator manual was developed and discussed among the investigators prior to finalisation.

#### Description of intervention

The intervention is at the practice level. It will be carried out over a six-month period, and consists of a training workshop, three practice visits by a facilitator in each state based in the Medicare Local, and three follow-up phone calls. The facilitators will be trained together using the facilitator manual and case examples from the pilot study.

**Figure 2:**
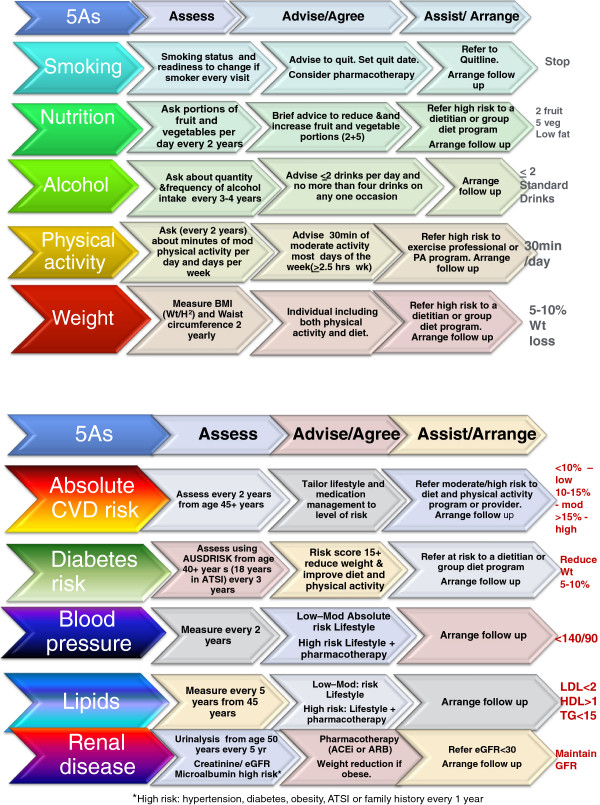
Reference guide: summary of guideline recommendations across the 5As.

The training workshop for GPs and PNs in each state on the prevention of CVD will be overseen and presented by a Chief or Associate Investigator, the Intervention Facilitator and a Division of General Practice/Medicare Local staff member. GPs and PNs from each of the intervention practices will attend the workshops. The format of the workshop will include an introductory presentation followed by case studies and role plays using simulated patients. The workshop introduces the 5As for smoking, nutrition, alcohol, physical activity, overweight, blood pressure, cholesterol, diabetes and absolute cardiovascular risk, and kidney disease. Guidelines that provide unambiguous advice and require fewer changes to practice are easier to implement. Thus, guideline recommendations have been synthesised across the 5As framework into a quick reference guide on two sides of an A4 sheet (Figure 2). These will be used as the basis for case discussion in the workshop.

A clinical audit will be provided to the GPs and PNs at baseline and 12 months for patients aged 40 to 69 without heart or kidney disease, stroke, or diabetes. This will focus on body mass index (BMI) and waist circumference, BP, lipids, fasting glucose, and absolute cardiovascular risk. This will include analysis of both the recording of behavioural and physiological risk factors and the risk factors themselves. Comparative data are provided from other practices together with a commentary prepared by an investigator in each state asking questions and suggesting areas for improvement.

**Figure 3:**
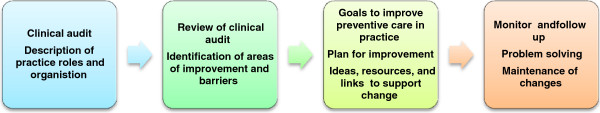
The intervention.

The three practice visits will occur at regular intervals within the six-month intervention period. Each practice will be asked to identify a prevention coordinator who is the key contact person for the research at the practice level (preferably the PN). Each practice visit will be of approximately 1 to 1.5 hours duration and include the GP(s), the PN, and possibly the practice manager. The practice visits will be conducted by the Intervention Facilitator and are designed to occur at regular intervals in order to build momentum and facilitate change at the practice level. They will discuss the provision of preventive care for each of the behavioural and physiological risk factors across the 5As (Figure 3).

Practice visit one will occur between one and four weeks post the training workshop. At this visit, the baseline audit results will be reviewed and two to three goals for improvement established. These goals might be to improve recording of certain risk factors, and intensify prescribing or advice given or referral to other services. Resources and local referral links will be provided and discussed at this initial visit as necessary.

Practice visit two will occur between three and four weeks after visit number one. The purpose of this visit will be to reflect on progress towards meeting the goals for improvement. Any barriers will be discussed and further resources and supports provided as needed. A particular focus in this visit will be on ensuring that preventive care is available for all patients including disadvantaged patients who may have low health literacy.

Practice visit three occurs between three and four weeks after visit number two. The purpose of this visit is to monitor improvements, workshop ways to overcome barriers and discuss how improvements might be maintained and incorporated as part of routine practice.

The Intervention Facilitator will conduct three follow-up and troubleshooting phone calls with the prevention coordinator. Each of the practice visits will be interspersed by a follow-up or trouble shooting phone call initiated by the intervention facilitator. Additional contact may be initiated by the prevention coordinator as required.

#### Participants and recruitment

Eight practices have been recruited by a primary care organisation (Medicare Local) in each of the four states (a total of 32 practices). Staff members from the Medicare Locals approached practices that met the eligibility criteria, such as having computerised medical records to enable an audit of medical records using the Pen Clinical Audit Tool (Pen CAT) [27] and a PN. Of the 47 practices who expressed interest in the study and who were subsequently visited, 32 agreed to participate, a response rate of 68%. Figure 2 shows the selection and randomisation process.

**Figure 4:**
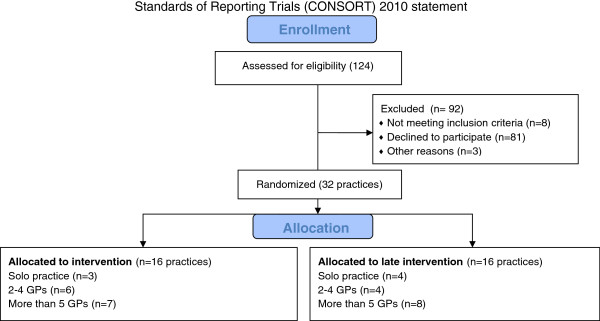
Participant Flow Diagram for the PEP Study consistent with the Consolidated Standards of Reporting Trials (CONSORT) 2010 statement.

In each practice, participants include practice staff and patients. As a minimum, at least one GP, one PN and one practice manager from each practice is involved in the study. However, in some of the group practices, two or more GPs are involved in the research. Of the 32 practices involved in the research, only seven are in solo practices whereas 15 of the practices employ five or more GPs and ten of the practices employ between two and four GPs (Figure 4).

The patients of the practice will be involved in the study in two ways. Firstly the records of all patients aged between 40 to 69 years who are active patients of the GPs who agree to participate in the study, and without known cardiac disease, stroke, or diabetes will be extracted in a de-identified audit at baseline and 12 months. In order to be seen as an active patient for the purpose of this study, patients must have visited their GP within the practice at least once in the last 12 months. Secondly, a random sample of 160 patients from each practice who consent and meet the eligibility criteria will be invited to complete the patient survey. To be eligible to participate in the study patients must have sufficient English and cognitive ability to understand the patient information letter, consent form, and written questionnaire.

#### Data collection procedures

**Figure 5:**
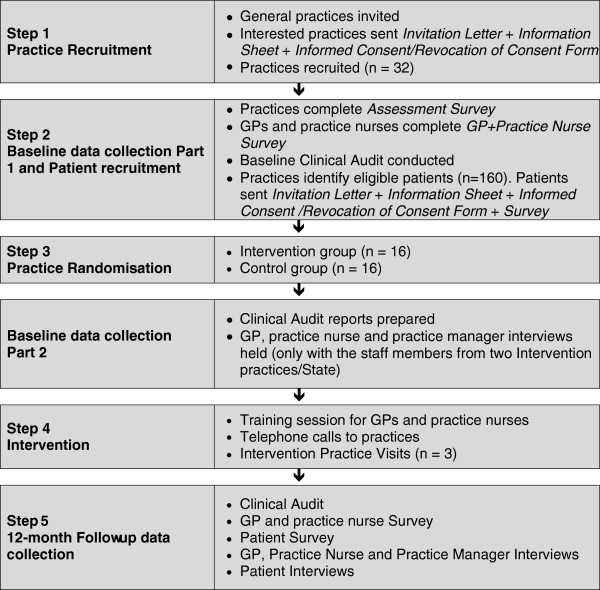
Step by step description of the PEP study.

Data will be collected from the practices, the practitioners and the patients for all 32 practices involved in the study (Figure 5). Data will be collected from practices by field research staff not involved in the conduct of the intervention. However, it may not be possible to fully blind them to allocation because practice staff may incidentally inform them during their visits.

The practice manager or the principal GP from each practice will be asked to complete a Practice Assessment Survey that collects descriptive information about the practice, including the location of the practice, number, type, and roles of staff members and information systems used. This information will assist us in understanding how the practice operates and therefore in identifying opportunities to facilitate preventive care. During the analysis phase of the research, the collation of practice information will enable us to examine whether any patterns of preventive care improvements emerge according to practice location, size, and teamwork arrangements.

Information will then be collected from the practitioners at baseline and again at 12 months. The GPs and PNs are asked to complete a survey that asks about their demographic characteristics and years in practice, and also how preventive care assessment and management is provided including frequency of assessment and management of the behavioural and physiological risk factors. This survey is based on questions from the Preventive Medicine Attitudes and Activities Questionnaire (PMAAQ) [28] used by us in previous research [29].

Patient information is also collected at baseline and again at 12 months to enable us to identify changes in the level of risk for CVD (Table 1). The medical record audit is conducted by the field research staff using the Pen CAT tool [27] The data extracted includes de-identified information on the recording and level of lifestyle and physiological risk factors, and the management of hypertension, dyslipidemia, diabetes, and CVD risk assessment. The patient survey is based on the NSW Health Survey [30] and previous research [31]. The survey includes questions about practice attendance, reported assessment and management of behavioural risk factors—smoking, nutrition, alcohol, physical activity and weight (SNAPW)—in general practice [7], attendances at other services as a result of referral from the practice or self-referral, self-reported fruit and vegetable intake [32], smoking, physical activity [33] and alcohol intake [34], and readiness for behaviour change (stage of change) for each SNAPW risk factor [35].

**Table 1:** Outcome Variables

**Category**	**Measures**	**Time Point**
Primary		
Recorded risk factors	Proportion of eligible patients with the following recorded	0 and 12 months
	· In the previous 24 months:-	
	o Blood pressure (in patients without hypertension)	
	o Weight (with height for BMI)	
	o Waist circumference	
	o Fasting blood glucose	
	oFasting Lipids	
	· Ever:-	
	o Smoking status	
	o Alcohol intake	
Recalled Advice	Proportion of eligible patients who were at risk who recalled being offered advice:	0 and 12 months
	· Diet (fruit and vegetables, low saturated fat)	
	· Physical activity	
	· Weight control	
Medication	Change in medications over previous 12 months	0 and 12 months
	· lipid lowering,	
	· antihypertensive	
Risk factors	Self assessed:	0 and 12 months
	· Physical activity score*	
	· Diet score**	
	· Smoking Status	
	· Alcohol intake (standard drinks per week)	
	Measured	
	· BMI	
	· waist circumference	
	· blood pressure	
	· lipids (TC, LDL-C, HDL-C, TG).	
Secondary		
GP or nurse preventive care	· Self reported assessment	0 and 12 months
	· Self reported advice	
GP or nurse	· attitudes to preventive care in general practice	0 and 12 months

* Physical activity level which combines assessment of duration of vigorous and moderate physical activity (scored 0-8, <4 considered at risk) [36].

** Serves of fruit and vegetables per day.

#### Qualitative study

A purposive sample of eight intervention practices (two in each state that includes a cross section of various practice sizes) will be qualitatively studied. This study will determine what individual and organisational factors explain the success of the PEP intervention and explore the role of the intervention facilitator in supporting practices to make improvements. The qualitative study is broadly informed by a variety of theories and frameworks that help us to understand the organizational routines and patterns of work [37,38], practice systems [39-41], and the way in which change is enacted and adapted in practices [42] and a framework for organization of care activities (Chronic Care Model) [43]. The study will include qualitative interviews conducted by the field researchers with practice staff involved in the intervention early and late in the intervention process. Other qualitative data will be collected from the intervention facilitators, including a diary of meeting and contacts with the practice, their notes on the implementation of the intervention, and an interview towards the end of the intervention period. Analysis will be conducted with the aid of NVivo using an approach previously trialed by Cohen et al. [44]. This will characterise narratives of the intervention at each study site looking for variation of key components, focus at each practice, and identifying factors which influence (or are perceived as influencing) the intervention.

#### Study size

The trial is being conducted in 32 practices. We estimate that the number of GPs and PNs recruited to the study will be 80.

We estimate that the records of at least 500 patients will be audited in each practice (i.e., 8,000 total). Assuming a design effect due to clustering of 1.8 based on previous studies [45], a sample of 188 patients in each group would have sufficient power (β = 0.8 and α = 0.05) to detect a 20% difference in the proportion of patients whose lifestyle and physiological risk factors are recorded. A sample size of 500 would have sufficient power to detect an effect size if 0.3 in recorded BMI (based on ICC = 0.047), LDL-Cholesterol (ICC 0.059), and systolic blood pressure after adjusting for clustering (ICC 0.062) [29,46,47].

It is estimated that at least 40 patients will be recruited to participate in the patient survey per practice or 640 patients in each arm of the trial. Allowing for 10% loss to follow up at 12 months, this will leave 36 patients per practice (576 in each group). Assuming a design effect due to clustering of two based on previous studies, a sample of 500 patients in each group would have sufficient power (β = 0.8 and α = 0.05) to detect a 15% difference in the proportion of patients offered education for diet or physical activity after adjusting for clustering (ICCs 0.051 and 0.035) and a 10% difference referral (ICCs 0.026 and 0.011). This sample size would have sufficient power to detect an effect size of 0.3 in serves of fruit and vegetables consumed (ICC 0.001) and physical activity scores (ICC 0.018) [33].

#### Statistical methods

We will examine the change of study variables within the intervention and control practices before and after interventions and compare the difference of outcomes between the two groups after adjusting for baseline differences. Primary analyses will be by intention to treat (patients and practices will be analysed as randomised, rather than by intervention actually received). We will analyse patient variables (risk behaviour, health service use, blood pressure, total cholesterol, HDL, LDL, General Practice Assessment Survey (GPAS) for within and between group differences using multilevel regression techniques adjusted for clustering of patients (level one) within practices (level two). The pre-randomisation value of each outcome will be used as lag covariates. Interactions as well as main effects will be tested. Secondary analysis will compare patients who were referred against those who were not, and defined as at risk. For cases lost to follow up at 12 months, we will conduct sensitivity analyses of the primary and secondary outcomes to determine the effect of their inclusion assuming no change in the outcome variables.

### Ethics

The study has been approved by the Royal Australian College of General Practitioners Human Research Ethics Committee and the Southern Adelaide Clinical Human Research Ethics Committee. This approval has been endorsed by the Human Research Ethics Committees at the University of New South Wales, Monash University, Bond and Queensland Universities. We obtained full informed written consent from participants.

### Project management

The study is led by a project management committee that comprises the investigators and the project coordinator, which meets bimonthly. Intervention- and data collection subcommittees meet as required. There are Chief Investigators (CIs) from each of the four states participating in the study who implement the study in each state together with the Field Research Officers and the Intervention Facilitators. The Field Research Officers are responsible for the data collection. Their roles include conducting the clinical audit and administering surveys and interviewing practice staff and patients. The Field Research Officers are based at the local University and do not interact directly with the intervention facilitators. The Intervention Facilitators are responsible for working with practices to facilitate improvements in preventive activities. Their roles include working with practices to set appropriate targets and goals, provide resources to assist the practices in meeting their goals, monitoring improvements, and identifying how changes and improvements can be maintained. It is necessary that the data collection and the facilitation roles are carried out by different staff members so as not to contaminate the results. The Intervention Facilitators are based in the local Division of General Practice or Medicare Local.

### Trial status

The trial is registered with the Australian Clinical Trials Registry ACTRN12612000578808.

The trial is underway with baseline data collection complete and the intervention commenced.

## Discussion

The PEP study aims to evaluate the effectiveness of an implementation strategy for cardiovascular preventive care guidelines in Australian general practice. This has the potential to address a significant evidence to practice gap. Although there is self-reported use of guidelines by most GPs, this does not mean that the guideline recommendations are routinely followed [48]. There are significant barriers to be overcome, and considerable variation exists between practices and practitioners in their readiness to implement evidence based care [49]. Effective practice interventions need to be tailored to the barriers and local context [50], be multifaceted [10], and involve the entire primary care team [51]. Thus, the implementation strategy has been designed to take into the context of individual practices and their staff with a flexible approach based on small group education, medical record audit, and practice facilitation. This is a unique approach in the Australian context and one that is also being explored overseas [52].

The complexities of applied research have led us to shift our focus from examining what works in preventing CVD generally, to focus more specifically on how primary care practices can be best supported in different contexts. By working with practices across four states in Australia, we hope to identify important characteristics and processes that can help generate and sustain a collaborative approach to preventing CVD. A key function of this partnership project is to ensure that the findings emerging from the research are communicated to a range of government and non-government organisations. Space has been allocated into the timeframe to enable potential implementation strategies and operational changes to be considered as part of the project dissemination process.

## Abbreviations

CVD: Cardiovascular disease; GP: General practitioner; NHMRC: National health and medical research council; PEP: Preventive evidence into practice (project); Pen CAT: Pen clinical audit tool; SNAPW: Smoking nutrition, alcohol, physical activity and weight.

## Competing interests

The authors declare that we have no competing interests.

## Authors’ contributions

MH, conceived of the study. All authors (except SP and YK) were involved in the design of the study and development of the proposal for funding. SP is the study coordinator. MH and JL wrote the initial draft. All authors have been involved in reviewing and editing the manuscript and read and approved the final draft.
